# Synergistic Effects of Non-Thermal Plasma Exposure Time and Drought on Alfalfa (*Medicago sativa* L.) Germination, Growth and Biochemical Responses

**DOI:** 10.3390/ijms27010330

**Published:** 2025-12-28

**Authors:** Mohamed Ali Benabderrahim, Imen Bettaieb, Valentina Secco, Hedia Hannachi, Ricardo Molina

**Affiliations:** 1 Arid and Oases Cropping Laboratory LR16IRA02, Arid Lands Institute (IRA), Medenine 4119, Tunisia; mohamedali.benabderrahim@fst.utm.tn (M.A.B.); imenbettaieb12@gmail.com (I.B.); 2 Department of Biology, Faculty of Sciences of Gabes (FSG), Erriadh City, Gabes 6072, Tunisia; 3 Department of Biological Chemistry, Plasma Chemistry Group, Institute of Advanced Chemistry of Catalonia (IQAC), Consejo Superior de Investigaciones Científicas (CSIC), Jordi Girona 18-26, 08034 Barcelona, Spain; vseccose8@alumnes.ub.edu; 4 Laboratory of Plant Productivity and Environmental Constraint (LR18ES04), Department of Biology, Faculty of Sciences of Tunis (FST), University Tunis El Manar, Tunis 2092, Tunisia; hedia.hannachi@fst.utm.tn

**Keywords:** alfalfa, NTP plasma, seed coat, germination, growth, drought, biochemicals

## Abstract

Applying non-thermal plasma (NTP) to seeds prior to sowing is recognized for its ability to enhance germination and promote plant growth. This study investigated the effects of NTP seed treatment on alfalfa seed surface characterization, germination, growth, and biochemical traits under varying water conditions. NTP modified seed surface properties by decreasing water contact angle, roughening the coat, and reducing O–H/N–H and C–H band intensities, while major functional groups remained intact. Short plasma exposures (<2 min) enhanced germination, whereas prolonged treatment (10 min) reduced viability, indicating embryo sensitivity. In pot experiments, both 1 and 5 min treatments improved fresh and dry weight, stem and root elongation, pigment accumulation, and protein content, particularly under normal and moderate water stress, while extended exposure (10 min) offered limited benefits and could be detrimental under severe drought. Root growth was most responsive, suggesting enhanced water and nutrient uptake. Plasma had modest effects on polyphenols and flavonoids but influenced early physiological responses and antioxidant activity. These findings highlight NTP as a promising seed priming tool to improve alfalfa performance, though further studies are needed to clarify the mechanisms and specific contributions of plasma components.

## 1. Introduction

Alfalfa (*Medicago sativa* L.) is one of the most important forage legumes worldwide due to its high protein content, strong regrowth capacity, and key role in sustainable agriculture. However, its establishment and early seedling growth are highly sensitive to water availability, particularly in arid and semi-arid regions where drought stress limits germination and stand uniformity. Seed germination under limited moisture is highly affected by seed coat permeability, imbibition kinetics, and early metabolic activation, making seed-enhancement technologies critical for improving field emergence under water stress [1].

Plasma, often referred to as the fourth state of matter, is naturally present in the universe (e.g., auroras), although it can also be artificially generated by means of an electrical discharge in a gas (e.g., neon tubes). Plasma is composed of a variety of reactive species, including radicals, electrons, ions, and neutral atoms, as well as UV–visible radiation. The modifications induced by these chemical species, such as oxidation processes and the formation of hydrophilic functional groups, are typically confined to only a few nanometres of the material surface and therefore do not alter the bulk properties of the treated material. Atmospheric non-thermal plasmas (NTPs) have recently emerged as an eco-friendly method for seed bio-activation, offering advantages such as improved wettability, surface etching, microbial decontamination, and modulation of physiological processes without chemical residues. Several studies have reported that NTP modifies seed surface chemistry by introducing oxygen-containing functional groups, reducing the contact angle, and enhancing water uptake [2,3]. These changes in seed surface can accelerate imbibition, promote enzymatic activation, and stimulate early germination dynamics, making NTP a promising tool for improving seed performance under adverse conditions [4]. Beyond its physical effects on the seed coat, NTP treatment has been shown to induce deeper physiological responses, including enhancement of antioxidant activity, modulation of metabolic pathways, and increased accumulation of phenolic compounds and flavonoids [5,6]. These biochemical defenses can improve seedling tolerance to oxidative stress generated during drought. Furthermore, improvements in seedling biomass, root elongation, and shoot growth after NTP exposure have been widely documented, suggesting that plasma-triggered signaling can influence early plant vigor and stress resilience [7].

Despite growing evidence of the benefits of cold plasma, its effects on alfalfa seeds under controlled water stress remain insufficiently explored, particularly in relation to multi-level responses spanning seed imbibition, surface morphology, germination performance, antioxidant capacity, and early growth. In addition, the interaction between plasma-induced surface modifications (e.g., contact angle, SEM microstructure) and biochemical responses during drought stress has not yet been fully characterized. Therefore, the present study aims to evaluate the effects of NTP treatment on alfalfa seeds, at different exposure time, subjected to different levels of water stress, by integrating analyses of seed germination, imbibition, contact-angle measurements, SEM imaging, early biomass accumulation (fresh and dry weight), and antioxidant-related biochemical parameters including proteins, polyphenols, flavonoids, and DPPH activity.

## 2. Results and Discussion

### 2.1. Alfalfa Seed Wettability

**Figure 1:**
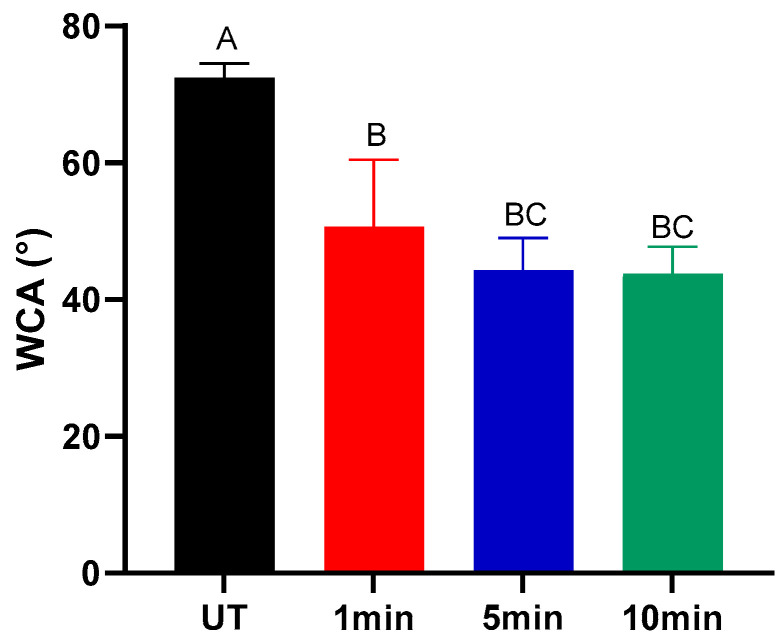
Water contact angle (WCA°) of untreated and NTP-treated alfalfa seeds. Different letters indicate significant differences between groups (*p* < 0.05).

NTP treatment induced a marked decrease in the water contact angle of alfalfa seeds (Figure 1), signifying increased surface hydrophilicity. Such a decrease is consistent after 5 min. The observed reduction in contact angle is attributed to plasma-mediated surface functionalization, wherein oxygen-based polar functional groups are incorporated onto the seed surface, significantly improving their affinity for water [8,9]. This enhanced wettability is a fundamental mechanism behind the physiological benefits of plasma treatment, as it facilitates faster and more uniform imbibition [10]. The results indicate that treatment duration modulates this effect, with significant changes occurring within the first minute of exposure. This enhanced wettability facilitates faster water uptake during imbibition, which is a critical first step in activating metabolic processes necessary for germination. The decrease in contact angle observed in plasma-treated seeds can be attributed to the generation of hydrophilic groups induced by the reactive species generated during the treatment. The optical emission spectra (OES) analysis confirms the presence of key reactive oxygen species (ROS) and reactive nitrogen species (RNS) (see Figure S1 in Supporting Information). Together, these results indicate that plasma-derived reactive species modify the seed coat chemistry, leading to enhanced surface hydrophilicity and a measurable reduction in contact angle.

### 2.2. Surface Structural Changes in Alfalfa Seeds Coats After NTP Exposure

#### 2.2.1. SEM

**Figure 2:**
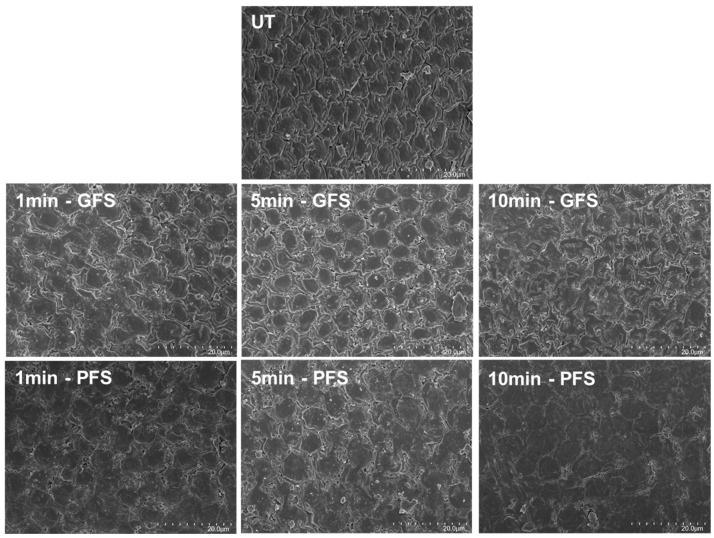
SEM micrographs of alfalfa seed coat at 2k magnification of untreated (UT) and NTP-treated seeds with different exposure times (1 min, 5 min, 10 min), showing progressive surface modifications. Both seed sides are shown: the plasma exposure (PFS: plasma-facing side) and the opposite side (GFS: reactor glass-facing side).

The SEM micrographs (Figure 2) illustrate the morphological changes on the seed coat surface of alfalfa following exposure to NTP for different durations (1 min, 5 min, and 10 min) compared with untreated seeds (UT). At 2k magnification, the overall morphology of the epidermal cells is clearly distinguishable, with well-defined cell outlines and visible intercellular spaces. The boundaries and arrangement of adjacent cells can also be readily observed. For the UT seeds, the epidermal cells of the seed coat surface appear smooth and intact. The structure shows a compact arrangement with well-preserved waxy layers, typical of intact seed coats that act as a protective barrier. Compared with UT, the surface of seeds treated for 1 min begins to show noticeable modifications; irregularities, slight erosion, and removal of surface debris are visible, indicating the initial etching effect of NTP, particularly in the plasma-facing side. The microstructure becomes rougher, suggesting that plasma starts breaking down the outer waxy cuticle. With an NTP treatment of 5 min, more pronounced changes appear: the surface is significantly roughened, with deeper cracks and etching. Plasma exposure clearly disrupts the seed coat integrity, creating micropores that may enhance water permeability and gas exchange. This structural modification reduces physical dormancy barriers. In addition, the intercellular space appears reduced after plasma treatment of seed coats, such as alfalfa, after all exposure time, but more pronounced after 10 min. The reduction in intercellular gaps observed in SEM images after plasma exposure could be explained by the removal of surface waxes and cuticular layers, combined with radical-induced weakening and partial collapse of the epidermal cell walls. At NTP treatment of 10 min, the surface is deeply altered, with large cracks, peeling layers, and erosion of the seed coat matrix. By using a SEM, many researchers reported the etching effect on the seed coat of wheat [11,12], barley [13], and nasturtium [14]. The observed etching can be attributed to the interaction between the NTP-specific chemicals and the energy distribution of the NTP with the seed surface coat [10]. Accordingly, in alfalfa, these structural modifications could cause enhancement of water uptake and improvement of germination, particularly at moderate NTP exposure time after 5 min. Our results align closely with previous findings on alfalfa seeds treated with non-thermal plasma [8], where plasma-induced structural and chemical modifications enhanced water uptake and germination at optimal doses, but excessive exposure led to surface over-etching and potential cellular damage, reducing germination rates. This supports the idea of a time-dependent response to NTP in alfalfa, where beneficial effects are achieved only within a specific treatment window. The observed alterations in plasma-treated seeds (surface etching and removal of waxy layers) could be directly related to improved water imbibition and can contribute to enhancing germination. This increased surface area can improve water retention and provide more sites for water entry, thereby promoting uniform and faster imbibition. Moreover, surface roughness may enhance the seed’s interaction with oxygen and other environmental cues, which are essential for initiating germination.

#### 2.2.2. ATR-FTIR

**Figure 3:**
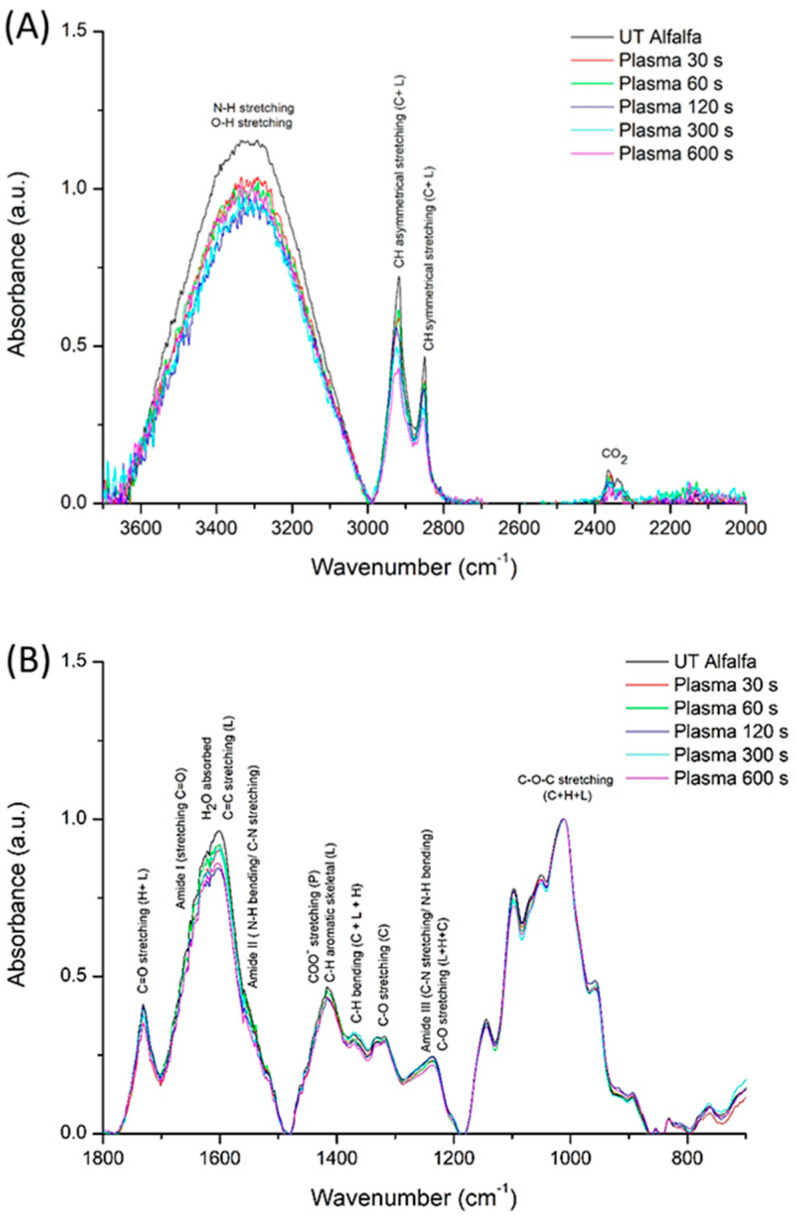
ATR-FTIR spectra of UT and plasma-treated alfalfa seeds at different exposure times in the wavenumber regions: (**A**) 3800–2600 cm^−1^ (**B**) 1800–600 cm^−1^. The side directly exposed to plasma was measured.

To detect chemical functional-group changes, FTIR is a valuable complementary technique for characterizing chemical changes on the seed-coat surface after plasma treatment [15]. Also, it confirms degradation or modification of waxes and cuticles. The ATR-FTIR spectra (average of the three replicates) of the UT and treated alfalfa seed cots are shown in Figure 3. The spectrum of the UT seeds is characterized by several peaks that can be attributed to major functional groups mainly associated with lipids, proteins, cellulose, hemicellulose, lignin, and adsorbed water. In the 1800–700 cm^−1^ region, no significant changes in band intensity are observed, except for a slight decrease in the band at 1635 cm^−1^, associated with adsorbed water, lignin C=C bonds, and Amide I and II vibrations [16,17,18,19]. In the high-wavenumber region (3600–2800 cm^−1^), all samples exhibit a broad absorption band centered around 3300–3400 cm^−1^, corresponding to O–H and N–H stretching vibrations. In alfalfa, the O–H groups come from adsorbed water, lignin (phenolic hydroxyls), and from carbohydrates (cellulose and hemicellulose have alcoholic –OH groups). The N–H signal (and associated amide bands) comes from peptide bonds of proteins [20]. Plasma-treated seeds show a clear decrease in the intensity of this band when measured immediately after treatment compared with the UT control, indicating water desorption during plasma exposure. This effect has also been reported in other seeds, such as wheat and nasturtium, and is similarly correlated with water loss induced by plasma exposure. On the other hand, the C–H stretching region (3000–2800 cm^−1^) exhibits relatively similar peak positions assigned to symmetric and asymmetric stretching of aliphatic groups of lipids, cellulose, and lignin [11,21]. A small and progressive decrease in intensity is observed with plasma exposures. This could be explained by little etching of surface lipid components as suggested by SEM observations (Figure 3), a phenomenon frequently reported in plasma-treated seeds and biomaterials [15]. These chemical changes in seed surfaces can further improve water absorption by increasing the hydrophilic character of the surface and may also influence enzymatic activities or alter the permeability of the seed coat, indirectly supporting radicle emergence and early growth.

### 2.3. Effect of NTP Exposure Time on Imbibition and Germination of Alfalfa Seeds

**Figure 4:**
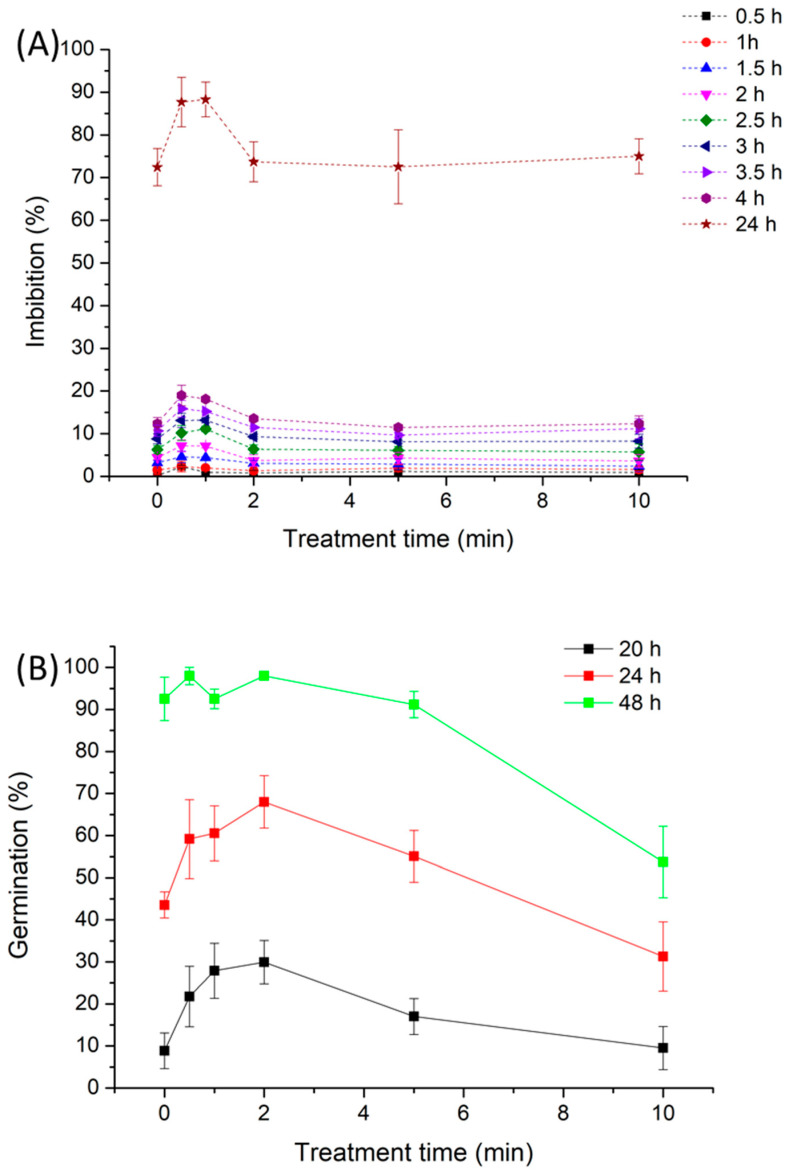
Imbibition (**A**) and germination (**B**) as a function of plasma treatment time for different imbibition periods of alfalfa seeds under laboratory conditions. Data points are connected for visual guidance only.

Figure 4 shows the imbibition and germination at different imbibition times (20, 24, and 48 h) of alfalfa seeds treated with NTP as a function of treatment time. Regarding seed germination (Figure 4b), the percentage of germinated seeds at 20 and 24 h increased significantly compared with untreated seeds for plasma treatment times of 2 min or less. Longer treatment times had a detrimental effect, with germination showing a decreasing trend, particularly for seeds treated for 10 min, where even lower germination percentages were observed at 24 h. Final germination at 24 h reached similarly high values (93–97%) for untreated seeds and seeds treated for short plasma durations (<2 min), whereas seeds treated for 10 min exhibited a drastic reduction in final germination, reaching approximately 50%. As water uptake of untreated and 10 min plasma-treated alfalfa seeds shows similar values at the different imbibition times, it is suggested that excessive plasma exposure could damage the alfalfa seed embryo, potentially rendering it non-viable for germination. Prolonging exposures may not only be redundant but could also risk over-etching the seed coat, potentially leading to cellular stress or viability loss [22]. Consequently, from both biological efficacy and practical application perspectives, 1 min appears to be an optimal NTP treatment duration for enhancing alfalfa seed germination.

### 2.4. Effects of NTP on Growth and Production of Alfalfa Plants Grown Under Different Field Capacity Levels

Table 1 shows the effect of NTP seed treatment and field capacity levels on alfalfa fresh weight, dry weight, and plant growth monitoring 45 days after sowing.

#### 2.4.1. Fresh Weight

Under good watering conditions (100%FC), all plants obtained from plasma-treated seeds (1, 5 min) show slightly higher fresh weight than untreated (UT). In contrast, the 10 min exposure shows a slight reduction in fresh mass of plants, suggesting overexposure may be detrimental. At moderate water stress (60%FC), plasma treatment significantly increases fresh weight, especially at 5 min (120.8 ± 30.2 mg/plant), compared with untreated (52.3 ± 10.8 mg/plant). This indicates plasma treatment can enhance growth under moderate water stress. Under high water stress (30% FC), fresh weight is generally lower, and plasma treatment does not significantly improve biomass. The reduction in 1, 5, and 10 min treatments suggests that severe drought stress limits the positive effect of plasma. As a result, we can conclude that plasma treatment enhances biomass, particularly under optimal and moderate water conditions. Similar effects were observed by [23], who reported that plasma-primed seeds improved seedling growth under moderate water stress.

#### 2.4.2. Dry Weight

As shown in Table 1, under well-watered conditions, plasma exposure had variable effects. Short treatments (1 min) maintained DW (dry weight) compared with untreated seeds (16.8 ± 2.9 mg/plant), but longer exposure (5–10 min) slightly increased DW (20.0 ± 12.4 and 19.5 ± 7.6 mg/plant, respectively). This suggests that under a non-limiting water supply, plasma does not highly enhance dry biomass. However, with moderate water deficit (60% FC), all plasma-treated seeds (1, 5, and 10 min) showed higher dry weight compared with untreated. This suggests that plasma priming may improve drought resilience by increasing the plants’ dry weight. In case of severe water deficit (30% FC), the plant dry weights were lower than those of the control and UT. The plasma treatments did not significantly alleviate the negative effect of severe drought, and in some cases (1 min, 5 min), DW remained very low. Consequently, it could be concluded that plasma priming appears to partially mitigate drought effects at moderate stress (60% FC), but under severe stress (30% FC), the benefit is minimal. Differently, [24] reported that by studying the dry weight of the shoot and root of oilseed rap treated by cold plasma, it was significantly increased compared with the drought-stressed seedlings. Also, [25] confirmed that plasma treatment could enhance the dry weight of radishes. It is clear that plasma performance depends on plant species and water conditions.

#### 2.4.3. Stem Elongation

The plasma treatments of seeds for 1 and 5 min significantly increased stem length (around 43–44 mm) compared with the untreated (~32 mm) at the control condition (100%FC). But 10 min exposure slightly reduces the effect. When plants were subjected to moderate water stress of 60%FC, the stem elongation increased with plasma treatments (1 and 5 min) from 25 mm (UT) to 38.5 mm (5 min). The effect of 10 min exposure time is slightly lower. Concerning the stress condition of 30%FC, stem elongation is limited (17 to 23 mm), with minor differences between plasma and untreated seeds. Water limitation likely dominates growth control in all plasma conditions. Consequently, plasma treatment stimulates stem elongation under favorable and moderate water conditions but cannot overcome severe drought. This aligns with findings reported by [26], showing that the DBD (Dielectric Barrier Discharge) plasma treatment could alleviate the adverse effects of drought stress on wheat seedling growth, and by [27], reporting that cold plasma seed treatment has demonstrated promise in improving various growth parameters in legumes.

#### 2.4.4. Root Elongation

As regards the roots elongation, under control conditions (100%FC), seed plasma treatments of 1 and 5 min significantly increased the root length (51 and 49.9 mm, respectively) compared with untreated (27 mm), indicating enhanced root development. As well, at 60% FC, plasma treatments at 5 and 10 min significantly improved the root length (58 and 62 mm, respectively) over UT (48.5 mm). Therefore, enhanced root systems under moderate stress can improve water uptake. At severe water stress (30%FC), the root growth shows minor improvement with 5 min plasma. So, the severe water stress limits root growth. Accordingly, plasma seed treatment successfully boosts root elongation, particularly under moderate stress, improving potential drought resilience. Similar observations were made on soybeans, showing plasma treatment of seeds developed longer roots [28]. Comparing the three growth traits, root growth responds most consistently to plasma treatment, suggesting enhanced water/nutrient uptake capacity. Also, the application of cold plasma for 1 or 5 min generally enhances all growth traits, but 10 min could sometimes reduce benefits, particularly in severe drought (30%FC).

### 2.5. Biochemical Composition

#### 2.5.1. Chlorophylls and Carotenoids

**Figure 5:**
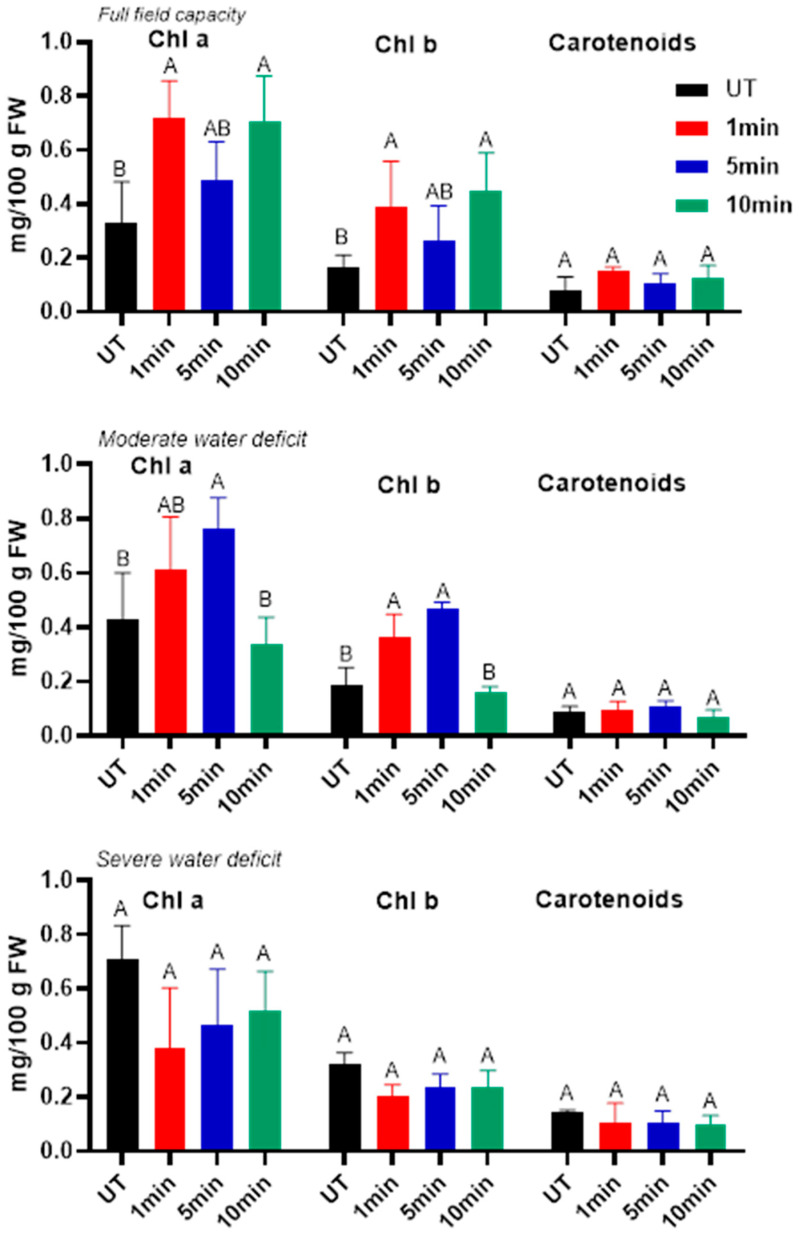
Chlorophylls and Carotenoids in alfalfa leaves at a stage of 45 days in different conditions of cold plasma exposure time and water field capacity. Different letters indicate significant differences between groups (*p* < 0.05).

Pigment profile analysis was performed to investigate the physiological responses of plants to plasma-induced seed priming under different water regimes (Figure 5). Results show that all pigments were affected depending on field capacity and plasma treatment duration. Notably, untreated plants (UT) exposed to 60% FC showed moderate increases in chlorophyll a, chlorophyll b, and carotenoids compared with full irrigation. Under severe drought (30% FC), chlorophyll a and b contents were dramatically enhanced, suggesting a protective response of the plant to abiotic stresses evolved to sustain photosynthetic activity when water availability is limited. Several studies have found that drought stress activates genes that encode proteins involved in chlorophyll metabolism and photosynthesis, leading to an increase in pigment concentration [29,30]. The increased level of carotenoid content was typically observed during drought, as these pigments act as non-enzymatic antioxidants, shielding chloroplasts from photooxidative damage and promoting plant defensive response to climatic variations [31]. Furthermore, the acquired results demonstrate various NTP treatment duration-specific responses. In fact, under full field capacity (100% Field Capacity: FC), plasma treatment induces an increase in all pigment contents compared with the untreated plants. In addition, plants exposed to 1 min and 10 min of NTP treatment had an approximately twofold increase in chlorophyll and carotenoid content compared with the untreated ones. In fact, in non-limiting water conditions, a brief exposure (1 min) effectively improves imbibition and initiates pigment biosynthesis mechanisms, whereas an extended treatment (10 min) may unexpectedly facilitate more efficient mobilization of seed reserve, resulting in a temporary rise in detectable pigment levels [32]. Although the mean pigment levels were higher under the 5 min plasma treatment compared with the untreated seeds, the difference was not statistically significant. Therefore, the 5 min exposure cannot be considered to have a detectable effect on pigment accumulation in the leaves. Under moderate water deficiency (60% FC), seeds exposed to plasma for 1 and 5 min had higher pigment levels than UT plants, suggesting that NTP initiated physiological priming that increased pigment retention under stress [33]. In contrast, the 10 min treatment reduced pigment concentrations. In the same way, [34] reported that prolonged cold plasma exposure elicited a negative physiological response, evidenced by a significant reduction in chlorophyll content in Norway spruce needles. During extreme drought (30% FC), all treatments showed a significant reduction in pigment contents compared with well-watered plants, suggesting that under severe water limitation, the protective and stimulatory effects of cold plasma become insufficient, particularly at 1 min exposure time. Furthermore, despite its low value, carotenoid content remains relatively stable after NTP exposure, particularly at 5 min. This indicates their important protective function in scavenging singlet oxygen while sustaining photosystem activity during stress.

#### 2.5.2. Proteins

**Figure 6:**
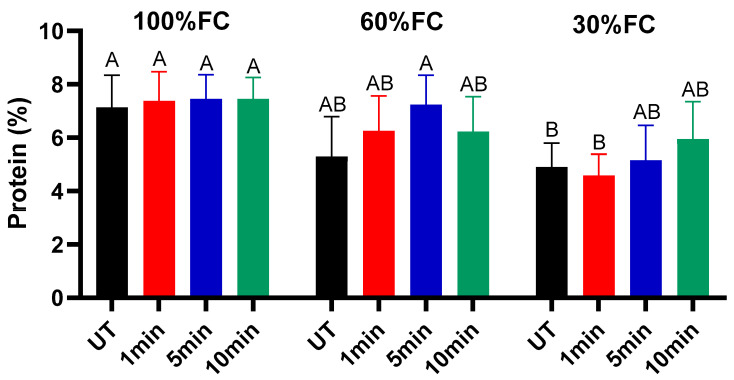
Proteins in alfalfa leaves at a stage of 45 days in different conditions of cold plasma exposure time and water field capacity. Different letters indicate significant differences between groups (*p <* 0.05).

The leaf protein content (% of DM) of alfalfa plants grown from seeds treated with NTP for varying exposure times is presented across three field capacity (FC) levels (Figure 6). While the effect of plasma exposure duration was not statistically significant, a significant interaction was observed between plasma treatment and soil water availability. Under well-watered conditions (100% FC), all NTP treatments led to a modest, non-significant increase in leaf protein content compared with the untreated control (UT). A similar trend was observed under moderate drought stress (60% FC), where NTP-treated seeds, particularly the 5 min exposure, yielded slightly higher protein levels than the control, though the effect was less pronounced than at 100% FC. In contrast, the most notable effects of NTP treatment emerged under severe drought stress (30% FC). As expected, severe water limitation caused a significant reduction in leaf protein content in control plants, reflecting a typical metabolic down-regulation under drought conditions [35]. However, this drought-induced decline was mitigated by NTP seed treatment. Protein levels in the 5 and 10 min NTP treatments were significantly higher than in the stressed control, indicating a protective effect of plasma priming. The observed enhancement in protein content, particularly under stress, suggests that NTP-induced seed surface activation and physiological priming improve metabolic efficiency during early development. This may lead to enhanced nitrogen assimilation and utilization during vegetative growth, a phenomenon supported by studies where plasma treatment upregulated nitrogen metabolism pathways [36]. Our findings demonstrate that NTP seed treatment slightly improves alfalfa, resulting in better retention of key metabolic processes like protein synthesis under drought conditions.

#### 2.5.3. Total Polyphenol Content (TPC) and Total Flavonoid Content (TFC)

**Figure 7:**
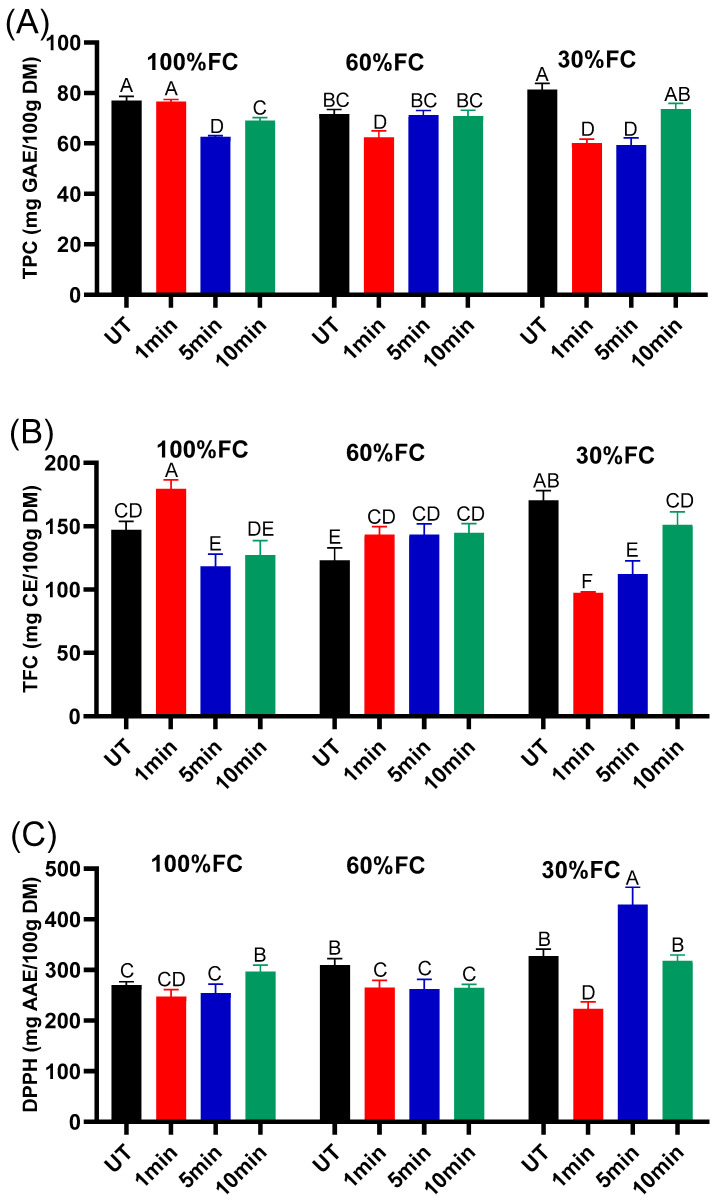
Total polyphenol content (**A**), total flavonoid content (**B**), and DPPH antioxidant capacity (**C**) in alfalfa leaf extracts at the stage of 60 days in different conditions of cold plasma exposure time and water field capacity. Different letters indicate significant differences between groups (*p <* 0.05).

Figure 7a illustrates the effect of NTP seed treatment and varying water stress conditions on the total polyphenol content (TPC) of plants aged 60 days. Accumulation of these antioxidant compounds is a common plant response to mitigate the increased oxidative stress caused by water deficit [37]. TPC in alfalfa leaves was only moderately affected by NTP exposure, while water availability remained the dominant factor shaping phenolic accumulation. Under 100% FC, plants grown from plasma-treated seeds showed TPC values similar (at 1 min) to or slightly lower (5 and 10 min) than the UT, indicating that plasma did not stimulate TPC accumulation under non-stressful conditions. This is consistent with reports showing that plasma effects on secondary metabolism are often modest when plants grow without abiotic stress [38]. At 60% FC, TPC remained almost stable across all plasma durations, suggesting that moderate drought did not interact strongly with plasma-induced priming. Under severe water deficit (30% FC), untreated plants exhibited the expected increase in phenolics associated with drought-driven antioxidant activation, whereas plasma-treated plants maintained comparable levels, particularly at 10 min. This trend aligns with studies showing that plasma can modulate oxidative responses without considerably increasing polyphenol synthesis unless strong oxidative stress is present [39]. In this study, cold plasma does not strongly upregulate polyphenol production in alfalfa, but it helps maintain stable levels across irrigation regimes.

Figure 7b shows the concentration of flavonoids for all conditions. Under 100% FC, the 1 min treatment led to TFC higher than that of the plants obtained from the UT seeds. But inverse results were obtained with 5 and 10 min. However, at medium stress conditions of 60%FC, the TFC slightly increased with plasma treatment compared with the control. This slight increase in TFC indicates that NTP enhanced the plant’s stress-induced metabolic response, stimulating flavonoid synthesis more effectively than in untreated plants [40]. In contrast, under severe water stress of 30%FC, the TFC, like TPC, decreased. Several drought studies show that flavonoid accumulation increases only up to a stress threshold, then declines as physiological damage accumulates [41]. The combined data obtained from TPC and TFC suggest that NTP priming has a higher effect on early physiological processes and primary metabolism than on later secondary metabolic pathways. To validate the hypothesis that plasma treatment triggers beneficial signaling rather than causing cellular damage, it would be essential to assess reactive oxygen species dynamics (H_2_O_2_, MDA, etc.) and oxidative stress markers at early seedling stages, enabling a clear distinction between adaptive signaling responses and detrimental oxidative injury.

#### 2.5.4. Antioxidant Activity

The variation of DPPH in alfalfa leaf extracts at a stage of 60 days in different experimental conditions is shown in Figure 7c. Cold plasma pre-treatment significantly affects DPPH radical-scavenging capacity in alfalfa leaves, with effects dependent on water field capacity. At normal water conditions and 60%FC, a slight decrease in DPPH was observed with NTP treatment, except for 10 min, where the antioxidant capacity increases. The strongest enhancement occurred under 30%FC conditions and 10 min plasma treatment. These findings support the hypothesis that NTP affects antioxidant metabolism and changes plant tolerance to environmental stress [42]. In fact, environmental stress indeed increases ROS, requiring strong antioxidant systems to maintain redox balance [43]. Consequently, this could be allowed by phenolic compounds and enzymatic antioxidants. We can conclude that NTP functions similarly to other priming strategies, potentially enhancing antioxidant capacity and protecting plants against ROS-mediated cellular damage, although further experiments are needed to determine the most suitable treatment conditions.

## 3. Materials and Methods

### 3.1. Plant Material

Alfalfa seeds (*Medicago sativa* L.), local accession from the oasis of Gabes, were obtained from farmers who harvested them in summer 2024 and were conserved in Institut des Régions Arides (IRA), Tunisia. The healthy seeds were selected for the control (Untreated with plasma; UT) and the three plasma treatments (1 min, 5 min, 10 min).

### 3.2. Plasma Treatment

**Figure 8:**
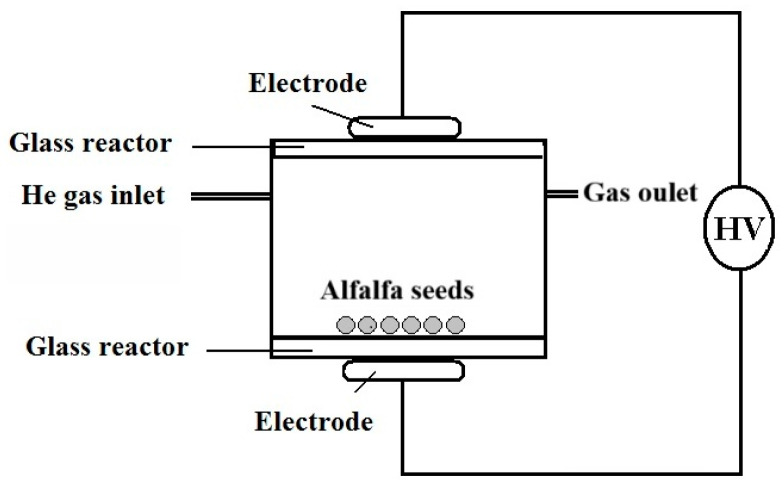
Scheme of the experimental set-up used for the atmospheric plasma treatment of alfalfa seeds. The device operated with helium gas at a discharge power of 30 W.

The Plasma treatment was performed in the Plasma Chemistry Laboratory, in the Department of Biological Chemistry, Plasma Chemistry Group, Institute of Advanced Chemistry of Catalonia (IQAC), Barcelona, Spain. The seeds were exposed to an atmospheric-pressure plasma generated in a DBD reactor assembled from simple borosilicate glass vessels and spacers (Figure 8) [14]. The device comprises two parallel metallic electrodes (45 mm in diameter) separated by a 12 mm gap, each one covered with a glass layer functioning as the dielectric barrier. Helium was selected as the primary feed gas to prevent arcing or intense microdischarges that could damage the seed coat. Its flow rate (5 Ln min^−1^) was regulated via a mass-flow meter and controller (Bronkhorst, Ruurlo, The Netherlands). Because the reactor is not hermetically sealed, ambient air mixes with helium, promoting the formation of reactive oxygen- and nitrogen-containing species that interact with seeds [44]. A 16 kHz alternating signal was supplied using a GF-855 function generator (Promax, L’Hospitalet de Llobregat, Spain), which fed a linear amplifier AG-1012 (T&C Power Conversion, Inc., Rochester, NY, USA). A matching network was connected to the amplifier output, followed by two transformers (HR-Diemen S.A., Sant Hipòlit de Voltregà, Spain), connected in a 180° phase-shifted configuration and therefore operating at floating potential, so that the resulting voltage applied to the electrodes was approximately doubled, reaching ~20 kV (see scheme in Figure 8). Alfalfa seeds (~2.5 g, visually intact) were placed on the lower glass plate covering the grounded electrode. The incident power was fixed at 30 W, and exposure times ranged from 0.5 min to 10 min. Optical emission spectroscopy (OES) data have been included in the Supporting Information to characterize the active species generated by our plasma device (see Figure S1, Supporting Information).

### 3.3. Seeds Surface Characterization (WCA, SEM, FTIR)

#### 3.3.1. Water Contact Angle (WCA)

Seed wettability was assessed by measuring the water contact angle on the seed surface. Because alfalfa seeds are small, a single droplet of distilled water (<1 µL) was carefully deposited onto each seed. Contact angles were then determined from the captured images using ImageJ software.

#### 3.3.2. Scanning Electron Microscopy (SEM)

The morphology of untreated and plasma-treated alfalfa seeds was examined using a FE-SEM HITACHI SU8600 scanning electron microscope (Hitachi High Technologies Co., Ltd., Tokyo, Japan) operated at an accelerating voltage of 5 kV at the Institute of Marine Sciences of the Spanish Research Council (CSIC). Prior to imaging, samples were coated with a ~5 nm iridium layer using a QUORUM 150T ES Plus sputter coater. SEM images were acquired from the seed surface directly exposed to plasma to enable consistent comparison among treatments.

#### 3.3.3. Fourier Transform Infrared Spectroscopy (FTIR) Analysis

ATR-FTIR analysis of the alfalfa seeds was carried out using a Nicolet AVATAR 360 spectrometer in the range of 400–4000 cm^−1^. Measurements were performed using the Smart iTR sampling Accessory (Thermo Scientific Inc., Waltham, MA, USA). Spectra were obtained from an average of 32 scans using a resolution of 4 cm^−1^. An advanced ATR correction algorithm (OMNIC 7.3 from Thermo Electron Corporation, Waltham, MA, USA) was used to correct the band intensity distortion, peak shifts, and polarization effects. All measurements were conducted in triplicate for each treatment. For consistency, each seed was positioned with the same face in contact with the ATR crystal, corresponding to the surface directly exposed to the plasma.

### 3.4. Experiments in Petri Dishes

#### 3.4.1. Seeds Water Imbibition

Water imbibition of untreated and plasma-treated alfalfa seeds was assessed by placing 0.5 g of seeds (≈200 seeds) in plastic Petri dishes containing two layers of filter paper (Whatman No. 1). This test was performed using 1.25 mL of distilled water on the upper paper and 0.75 mL on the lower paper, and the dishes were incubated at 25 °C. Seeds removed from the Petri dishes were carefully dried with paper towels, weighed on a digital scale, and returned for further imbibition. Water uptake was monitored at 30 min intervals over a 4 h period and again after 24 h. For each time point, three replicates were used. The percentage of water absorption was calculated using the following formula:

Imbibition (%) = (Wf − Wi) 100Wi

where Wf and Wi are the seed weight after and before the water immersion, respectively.

#### 3.4.2. Seeds Germination

Seed germination was carried out under the same experimental conditions described for the water imbibition assay. For each treatment, 49 seeds were placed in each Petri dish on Whatman paper n°1 and incubated at 25 °C. To ensure uniform moisture, 0.75 mL of water was added to the upper paper and 0.75 mL to the lower paper. Germination was monitored at 20, 24, and 48 h after sowing, and a seed was considered germinated when the radicle reached at least 2 mm in length. Three biological replicates were performed per treatment. Germination percentage was calculated using the following formula:

Germination (%) = (Number of germinated seeds/Total number of seeds) × 100

### 3.5. Experiment in Pots Under Different Field Capacities

A pot experiment was conducted under controlled growth-chamber conditions at the Institut des Régions Arides (IRA), Gabès, Tunisia. Thirty-six pots were filled with a soil mixture consisting of 50% sand and 50% organic compost, and thirty seeds were sown in each pot. The experiment followed a factorial design with three field-capacity levels (100% FC, 60% FC, and 30% FC) and four seed treatments (UT, 1 min, 5 min, and 10 min), each treatment combination being replicated three times. Irrigation with a nutrient solution was applied every two days according to the assigned field-capacity level of each pot.

### 3.6. Biomass Production and Growth

#### 3.6.1. Fresh and Dry Weights

Fresh and dry biomass were assessed 45 days after sowing. For each pot (biological replicate), four plants were randomly selected as subsamples and harvested, resulting in a total of twelve plants per treatment. Fresh weight was measured immediately after collecting using an analytical balance (FW; g/plant). The samples were then placed in aluminum envelopes and oven-dried at 70 °C until a constant weight was reached, after which the dry weight was recorded (DW; g/plant).

#### 3.6.2. Stem and Roots Length

Stem and root lengths were measured on fresh plants 45 days after sowing. From each pot, four plants used for fresh weight determination were carefully separated into shoots and roots. The lengths of stems and roots were measured from digital images using ImageJ software (version 1.53, National Institutes of Health, Bethesda, MD, USA), and all measurements were recorded in millimeters.

### 3.7. Biochemical and Antioxidant Analyses

#### 3.7.1. Chlorophylls and Carotenoids

Chlorophyll a, chlorophyll b, and total carotenoids were determined on fresh leaves. Four biological replicates were performed for each treatment. Approximately 0.2–0.5 g of leaf tissue was collected from each plant and homogenized in 80% acetone. The homogenate was centrifuged at 5000× *g* for 10 min, and the supernatant was collected. Absorbance of the extract was measured at 663, 645, and 470 nm using a spectrophotometer. Chlorophyll a, chlorophyll b, and total carotenoid contents were calculated according to standard equations and expressed on a fresh weight basis (mg 100 g^−1^ FW).

Chl a (mg/L) = 12.7 × A_663_ − 2.69 × A_645_

Chl b (mg/L) = 22.9 × A_645_ − 4.68 × A_663_

Carotenoids (mg/L) = [A470 − (0.026 × Chl a + 0.426 × Chl b)]/0.00802

#### 3.7.2. Protein Content

Total soluble protein content was determined in alfalfa leaves collected on day 60 after sowing and dried to constant weight. Approximately 0.5 g of dried leaf tissue per plant was used for analysis. Protein content was measured using the Kjeldahl method, which involves digestion of the sample in concentrated sulfuric acid with a catalyst, followed by distillation and titration to determine total nitrogen. Protein content was calculated by multiplying the measured nitrogen content by a conversion factor of 6.25 and expressed on a dry weight basis (% DW)

#### 3.7.3. Total Polyphenol Contents (TPC) & Total Flavonoids Contents (TFC)

Total polyphenol content (TPC) and total flavonoid content (TFC) were determined on dried alfalfa plants collected on day 60 after sowing. Approximately 0.5 g of dried sample was extracted with ethanol–water (1:1) under continuous shaking for 24 h at room temperature. The extracts were filtered, and TPC was measured using the Folin–Ciocalteu reagent, with results expressed as mg gallic acid equivalents per 100 g of dry weight (mg GAE 100 g^−1^ DW). TFC was determined using the aluminum chloride colorimetric method, with results expressed as mg catechin equivalents per 100 g of dry weight (mg CE 100 g^−1^ DW). Absorbance readings for both assays were performed using a spectrophotometer at the appropriate wavelengths.

#### 3.7.4. Antioxidant Capacity

Antioxidant capacity was determined on dried alfalfa plants collected on day 60 after sowing using the DPPH (2,2-diphenyl-1-picrylhydrazyl) radical scavenging assay. The same extract as TFC was filtered, and 0.2 µL of extract was mixed with 180 µL of DPPH solution (0.1 mM in ethanol). The mixture was incubated in the dark at room temperature for 30 min, and absorbance was measured at 517 nm using a spectrophotometer. The antioxidant activity was calculated as the percentage of DPPH radical scavenging and expressed relative to the standard antioxidant ascorbic acid, per 100 g of dry weight (mg AAE 100 g^−1^ DW)

### 3.8. Statistical Analysis

All experiments were conducted in a completely randomized design with three or four replicates per treatment. Data are presented as mean ± standard deviation (SD). Statistical analyses were performed using XLSTAT (version 2019.4.1, Addinsoft, Paris, France). Significant difference test for multiple comparisons was considered at the level of *p* < 0.05. Graphs and figures were prepared using GraphPad Prism (version 9.5, GraphPad Software, San Diego, CA, USA).

## 4. Conclusions

In this work, we focused on the study of germination, growth, and biochemical attributes of alfalfa under different NTP plasma treatment of seeds and water conditions. Seed surface characterization measurements indicated that the NTP duration decreased water contact angle, gradually roughened the seed coat, creating etching, and reduced O–H/N–H and C–H band intensities due to water loss and minor lipid etching, while major functional groups remain largely unchanged. In addition, NTP plasma treatment of alfalfa seeds for short durations (<2 min) enhances germination, while prolonged exposure (10 min) significantly reduces germination despite similar water uptake, likely due to embryo damage of some seeds. Thus, a 1 min NTP treatment is suggested as optimal for improving germination without harming seed viability. Regarding the pots experiment, NTP plasma seed treatment enhances alfalfa growth, with 1–5 min exposures increasing fresh and dry weight, stem, and root elongation, particularly under normal conditions and moderate water stress (60% FC) conditions. Longer exposure (10 min) shows reduced or negligible benefits and may even be harmful under optimal or severe drought conditions. Nevertheless, root growth responds most consistently to plasma, suggesting improved water and nutrient uptake and potential drought resilience. As regards pigment accumulation, NTP seed treatment enhances chlorophyll and carotenoid content under normal and moderate water conditions, while under severe drought, pigment accumulation declines, highlighting carotenoids’ protective role in stress tolerance. NTP seed treatment slightly increases leaf protein content in alfalfa, with the most pronounced effect under severe drought (30% FC). This suggests that plasma enhances nitrogen assimilation, supporting protein retention during vegetative growth. Nevertheless, it has a limited effect on total polyphenol and flavonoid accumulation in alfalfa and strongly influences early physiological responses and antioxidant activity (DPPH). Further research is required to distinguish the effects of the various plasma components on seeds and to clarify the underlying mechanisms, enabling more precise control over the results.

## Figures and Tables

**Table 1:** Effect of cold plasma seed treatment and field capacities levels on alfalfa fresh weight and plant growth (45 days). Different letters indicate significant differences between treatments.

	Plasma	FW (mg/Plant)	DW (mg/Plant)	SL (mm)	RL (mm)
Full field capacity (100% FC)	UT	77.0 ± 7.5 a	16.8 ± 2.9 a	31.8 ± 11.3 b	27.4 ± 1.9 c
	1 min	78.8 ± 8.7 a	17.0 ± 6.7 a	42.9 ± 4.0 a	51.0 ± 8.0 ab
	5 min	78.0 ± 36.2 a	20.0 ± 12.4 a	44.4 ± 10.3 a	49.9 ± 9.2 ab
	10 min	64.3 ± 12.3 ab	19.5 ± 7.6 a	34.6 ± 8.7 ab	46.6 ± 3.0 b
Moderate water deficit (60% FC)	UT	52.3 ± 10.8 b	13.3 ± 5.0 b	25.4 ± 2.2 b	48.5 ± 8.5 ab
	1 min	94.8 ± 25.0 a	29.5 ± 10.1 a	33.5 ± 1.6 ab	40.9 ± 2.8 b
	5 min	120.8 ± 30.2 a	31.3 ± 4.6 a	38.5 ± 9.6 a	58.0 ± 7.1 a
	10 min	82.8 ± 39.4 a	23.0 ± 12.8 ab	32.9 ± 10.9 ab	62.5 ± 24.0 a
Severe water deficit (30% FC)	UT	65.5 ± 18.0 ab	20.0 ± 6.0 a	20.1 ± 6.7 ab	49.9 ± 7.2 a
	1 min	48.5 ± 25.6 b	14.5 ± 5.8 ab	17.4 ± 2.9 b	45.3 ± 2.2 ab
	5 min	43.8 ± 18.4 b	13.0 ± 7.0 ab	22.7 ± 5.3 a	52.0 ± 7.7 a
	10 min	44.3 ± 4.2 b	19.3 ± 5.1 a	20.9 ± 3.3 ab	40.8 ± 1.5 b

Values are presented as mean ± SD; values with different letters indicate significant differences between groups (*p* < 0.05). FW: fresh weight, DW: dry weight, SL: Stem length, RL: root length.

## Data Availability

All data collected during the studies are included in this paper.

## Supplementary Materials

The following supporting information can be downloaded at https://www.mdpi.com/article/10.3390/ijms27010330/s1.

## Abbreviations

The following abbreviations are used in this manuscript:

NTP	Non-thermal plasma
SEM	Scanning Electron Microscopy
FTIR-ATR	Fourier Transform Infrared Spectroscopy-Attenuated Total Reflectance
WCA	Water contact angel
FC	Field capacity
TPC	Total polyphenol contents
TFC	Total flavonoids contents (TFC)

## References

[1] Wang R., Yang Y., Wang X., Li J., Gao Y., Huang H., Zhou Z., Wang P., Zhao L. (2025). Response of seed germination and seedling growth of perennial ryegrass (*Lolium perenne* L.) to drought, salinity, and pH in Karst regions. Sci. Rep..

[2] Bormashenko E., Grynyov R., Bormashenko Y., Drori E. (2012). Cold radiofrequency plasma treatment modifies wettability and germination speed of plant seeds. Sci. Rep..

[3] Sivachandiran L., Khacef A. (2017). Enhanced seed germination and plant growth by atmospheric pressure cold air plasma: Combined effect of seed and water treatment. RSC Adv..

[4] Fu Y., Ma L., Li J., Hou D., Zeng B., Zhang L., Liu C., Bi Q., Tan J., Yu X. (2024). Factors influencing seed dormancy and germination and advances in seed priming technology. Plants.

[5] Kumar S.P., Chintagunta A.D., Lichtfouse E., Naik B., Kumari K., Kumar S. (2022). Non-thermal plasmas for disease control and abiotic stress management in plants. Environ. Chem. Lett..

[6] Sultan S.M.E., Yousef A.F., Ali W.M., Mohamed A.A.A., Ahmed A.-R.M., Shalaby M.E., Teiba I.I., Hassan A.M., Younes N.A., Kotb E.F. (2024). Cold atmospheric plasma enhances morphological and biochemical attributes of tomato seedlings. BMC Plant Biol..

[7] Benabderrahim M.A., Bettaieb I., Rejili M. (2025). Boosting seed performance with cold plasma. Appl. Sci..

[8] Holc M., Gselman P., Primc G., Vesel A., Mozetič M., Recek N. (2022). Wettability and water uptake improvement in plasma-treated alfalfa seeds. Agriculture.

[9] Mohajer M.H., Monfaredi M., Rahmani M., Martami M., Razaghiha E., Mirjalili M.H., Hamidi A., Ghomi H.R. (2024). Impact of dielectric barrier discharge plasma and plasma-activated water on cotton seed germination and seedling growth. Heliyon.

[10] Starič P., Vogel-Mikuš K., Mozetič M., Junkar I. (2020). Effects of nonthermal plasma on morphology, genetics and physiology of seeds: A review. Plants.

[11] Molina R., Lalueza A., López-Santos C., Ghobeira R., Cools P., Morent R., De Geyter N., González-Elipe A.R. (2021). Physicochemical surface analysis and germination at different irrigation conditions of DBD plasma-treated wheat seeds. Plasma Process. Polym..

[12] Li Y., Wang T., Meng Y., Qu G., Sun Q., Liang D., Hu S. (2017). Air atmospheric dielectric barrier discharge plasma induced germination and growth enhancement of wheat seed. Plasma Chem. Plasma Process..

[13] Park Y., Oh K.S., Oh J., Seok D.C., Kim S.B., Yoo S.J., Lee M.J. (2018). Biological effects of surface dielectric barrier discharge on seed germination and plant growth with barley. Plasma Process. Polym..

[14] Molina R., López-Santos C., Gómez-Ramírez A., Vílchez A., Espinós J.P., González-Elipe A.R. (2018). Influence of irrigation conditions in the germination of plasma-treated Nasturtium seeds. Sci. Rep..

[15] Waskow A., Howling A., Furno I. (2021). Advantages and limitations of surface analysis techniques on plasma-treated Arabidopsis thaliana seeds. Front. Mater..

[16] Canteri M.H., Renard C.M., Le Bourvellec C., Bureau S. (2019). ATR-FTIR spectroscopy to determine cell wall composition: Application on a large diversity of fruits and vegetables. Carbohydr. Polym..

[17] Heredia-Guerrero J.A., Benítez J.J., Domínguez E., Bayer I.S., Cingolani R., Athanassiou A., Heredia A. (2014). Infrared and Raman spectroscopic features of plant cuticles: A review. Front. Plant Sci..

[18] Lei Y., Hannoufa A., Christensen D., Shi H., Prates L.L., Yu P. (2018). Molecular structural changes in alfalfa detected by ATR-FTIR spectroscopy in response to silencing of TT8 and HB12 genes. Int. J. Mol. Sci..

[19] Luan X., Song Z., Xu W., Li Y., Ding C., Chen H. (2020). Spectral characteristics on increasing hydrophilicity of alfalfa seeds treated with alternating current corona discharge field. Spectrochim. Acta A Mol. Biomol. Spectrosc..

[20] Puițel A.C., Bârjoveanu G., Balan C.D., Nechita M.T. (2025). Medicago sativa stems—A multi-output integrated biorefinery approach. Polymers.

[21] Ramlath K., Sajna P., Nusrath P., Rajesh C. (2023). Isolation and characterisation of cellulose fibre from Pennisetum polystachion and its application in biocomposites with EPDM rubber. Cellul. Chem. Technol..

[22] Tomeková J., Kyzek S., Medvecká V., Gálová E., Zahoranová A. (2020). Influence of cold atmospheric pressure plasma on pea seeds: DNA damage of seedlings and optical diagnostics of plasma. Plasma Chem. Plasma Process..

[23] Guo Q., Wang Y., Zhang H., Qu G., Wang T., Sun Q., Liang D. (2017). Alleviation of adverse effects of drought stress on wheat seed germination using atmospheric dielectric barrier discharge plasma treatment. Sci. Rep..

[24] Ling L., Jiangang L., Minchong S., Chunlei Z., Yuanhua D. (2015). Cold plasma treatment enhances oilseed rape seed germination under drought stress. Sci. Rep..

[25] Matra K. (2016). Non-thermal plasma for germination enhancement of radish seeds. Procedia Comput. Sci..

[26] Jiang J., He X., Li L., Li J., Shao H., Xu Q., Ye R., Dong Y. (2014). Effect of cold plasma treatment on seed germination and growth of wheat. Plasma Sci. Technol..

[27] Abeysingha D.N., Dinesh S., Kottage S.M., Chen L., Roopesh M.S., Thilakarathna M.S. (2025). Effects of cold plasma seed treatment on pea (*Pisum sativum* L.) plant performance under drought and well-watered conditions. PLoS ONE.

[28] Ling L., Jiafeng J., Jiangang L., Minchong S., Xin H., Hanliang S., Yuanhua D. (2014). Effects of cold plasma treatment on seed germination and seedling growth of soybean. Sci. Rep..

[29] Shan D., Wang C., Song H., Bai Y., Zhang H., Hu Z., Wang L., Shi K., Zheng X., Yan T. (2021). The MdMEK2–MdMPK6–MdWRKY17 pathway stabilizes chlorophyll levels by directly regulating MdSUFB in apple under drought stress. Plant J..

[30] Zhang C., Shi S., Wang B., Zhao J. (2018). Physiological and biochemical changes in drought-tolerant alfalfa (*Medicago sativa* L.) varieties under PEG-induced drought stress. Acta Physiol. Plant..

[31] Medyouni I., Zouaoui R., Rubio E., Serino S., Ahmed H.B., Bertin N. (2021). Effects of water deficit on leaves and fruit quality during tomato development. Food Sci. Nutr..

[32] Waskow A., Howling A., Furno I. (2021). Mechanisms of plasma-seed treatments as a potential seed processing technology. Front. Phys..

[33] Saini R., Das R., Adhikary A., Kumar R., Singh I., Nayyar H., Kumar S. (2022). Drought priming induces chilling tolerance and improves reproductive functioning in chickpea (*Cicer arietinum* L.). Plant Cell Rep..

[34] Čėsnienė I., Čėsna V., Mildažienė V., Miškelytė D., Vaitiekūnaitė D., Sirgedaitė-Šėžienė V. (2025). The impact of seed treatment with cold plasma on antioxidants, sugars, and pigments in needles of Norway spruce is genotype-dependent. Plants.

[35] Farooq M., Wahid A., Kobayashi N.S.M.A., Fujita D.B.S.M.A., Basra S.M. (2009). Plant drought stress: Effects, mechanisms and management. J. Sustain. Agric..

[36] Mildaziene V., Ivankov A., Sera B., Baniulis D. (2022). Biochemical and physiological plant processes affected by seed treatment with non-thermal plasma. Plants.

[37] Jan R., Asaf S., Numan M., Lubna, Kim K.M. (2021). Plant secondary metabolite biosynthesis and transcriptional regulation in response to biotic and abiotic stress conditions. Agronomy.

[38] Priatama R.A., Pervitasari A.N., Park S., Park S.J., Lee Y.K. (2022). Current advancements in the molecular mechanism of plasma treatment for seed germination and plant growth. Int. J. Mol. Sci..

[39] Konchekov E.M., Gusein-Zade N., Burmistrov D.E., Kolik L.V., Dorokhov A.S., Izmailov A.Y., Shokri B., Gudkov S.V. (2023). Advancements in plasma agriculture: A review of recent studies. Int. J. Mol. Sci..

[40] Patil J.R., Mhatre K.J., Yadav K., Yadav L.S., Srivastava S., Nikalje G.C. (2024). Flavonoids in plant–environment interactions and stress responses. Discov. Plants.

[41] Mehravi S., Hanifei M., Gholizadeh A., Khodadadi M. (2023). Water deficit stress changes in physiological, biochemical and antioxidant characteristics of anise (*Pimpinella anisum* L.). Plant Physiol. Biochem..

[42] Han B., Yu N.N., Zheng W., Zhang L.N., Liu Y., Yu J.B., Zhang Y.Q., Park G., Sun H.N., Kwon T. (2021). Effect of non-thermal plasma (NTP) on common sunflower (*Helianthus annuus* L.) seed growth via upregulation of antioxidant activity and energy metabolism-related gene expression. Plant Growth Regul..

[43] Das K., Roychoudhury A. (2014). Reactive oxygen species (ROS) and response of antioxidants as ROS-scavengers during environmental stress in plants. Front. Environ. Sci..

[44] Molina R., Ligero C., Jovančić P., Bertran E. (2013). In situ polymerization of aqueous solutions of NIPAAm initiated by atmospheric plasma treatment. Plasma Process. Polym..

