# Supplementary material for: Synergistic Effects of Non-Thermal Plasma Exposure Time and Drought on Alfalfa (Medicago sativa L.) Germination, Growth and Biochemical Responses
Source: Int J Mol Sci. 2025 Dec 28;27(1):330. doi: 10.3390/ijms27010330 (PMC12785691; doi:10.3390/ijms27010330)
Supplement: Supplementary file 1 [file ijms-27-00330-s001.zip › ijms-4042701-supplementary.pdf]

Supporting information

OES analysis was used to characterize the plasma active species. Light was collected through an optical fiber located in front of a reactor quartz window. The fiber was connected to a Black Comet spectrometer (Stellarnet, USA) with concave gratings. Spectra were recorded in the UV–VIS wavelength range (190–850 nm) with an integration time of 4 s. the OES spectrum recorded with Alfalfa seeds present shows the disappearance of the atomic oxygen emission line that is otherwise seen in the seed-free plasma spectrum, indicating a consumption or quenching of O species in the presence of seeds. The absence of the O emission suggests that interactions with seed surface moisture and desorbed water preferentially drive dissociation pathways (such as enhanced OH formation) at the expense of free atomic oxygen, consistent with the hypothesis that seed water content alters the plasma chemistry.

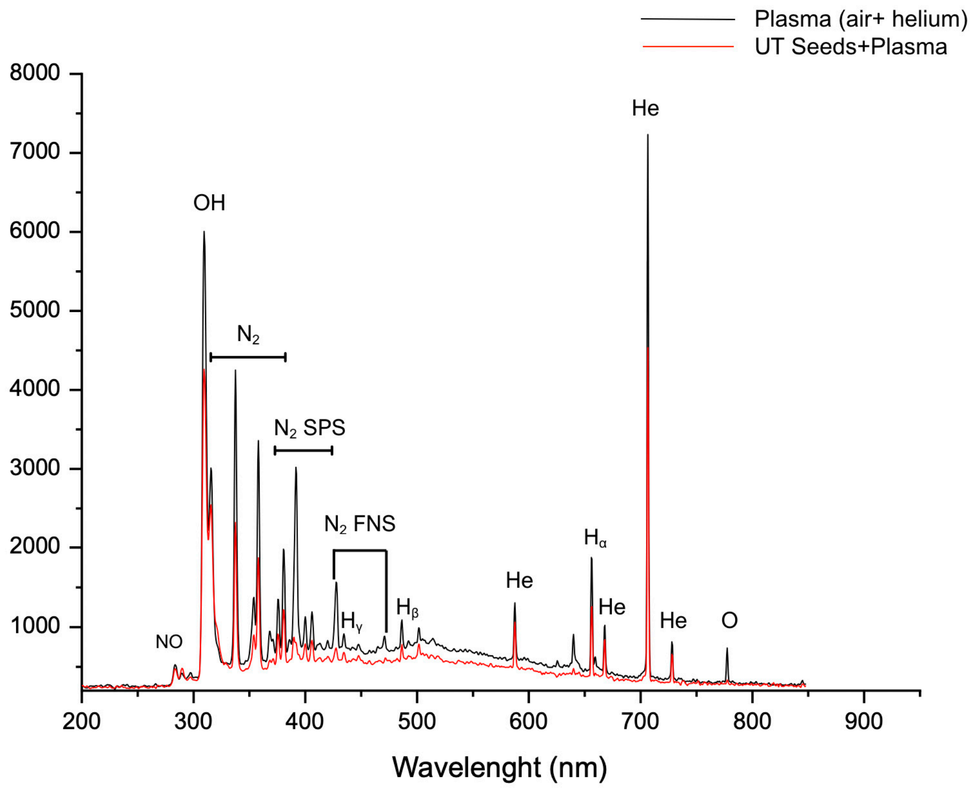

Figure S1. Optical emission spectra of the plasma generated showing the mean reactive species.
